# Myco-fabricated ZnO nanoparticles ameliorate neurotoxicity in mice model of Alzheimer’s disease via acetylcholinesterase inhibition and oxidative stress reduction

**DOI:** 10.1007/s10534-023-00525-6

**Published:** 2023-08-09

**Authors:** Hanan A. Abd Elmonem, Reham M. Morsi, Doaa S. Mansour, El-Sayed R. El-Sayed

**Affiliations:** 1 Biological Applications Department, Nuclear Research Center, Egyptian Atomic Energy Authority, Cairo, Egypt; 2 Plant Research Department, Nuclear Research Center, Egyptian Atomic Energy Authority, Cairo, Egypt

**Keywords:** Neurotoxicity, Alzheimer’s disease, ZnO nanoparticles, Mice, Myco-fabrication

## Abstract

Alzheimer’s disease (AD) is one of the primary health problems linked to the decrease of acetylcholine in cholinergic neurons and elevation in oxidative stress. Myco-fabrication of ZnO-NPs revealed excellent biological activities, including anti-inflammatory and acetylcholinesterase inhibitory potentials. This study aims to determine if two distinct doses of myco-fabricated ZnO-NPs have a positive impact on behavioral impairment and several biochemical markers associated with inflammation and oxidative stress in mice that have been treated by aluminum chloride (AlCl_3_) to induce AD. Sixty male mice were haphazardly separated into equally six groups. Group 1 was injected i.p. with 0.5 ml of deionized water daily during the experiment. Mice in group 2 received AlCl_3_ (50 mg/kg/day i.p.). Groups 3 and 4 were treated i.p. with 5 and 10 mg/kg/day of ZnO-NPs only, respectively. Groups 5 and 6 were given i.p. 5 and 10 mg/kg/day ZnO-NPs, respectively, add to 50 mg/kg/day AlCl_3_. Results showed that the AlCl_3_ caused an increase in the escape latency time and a reduction in the time spent in the target quadrant, indicating a decreased improvement in learning and memory. Moreover, acetylcholinesterase enzyme (AChE) activity and malondialdehyde (MDA), tumor necrosis factor-alpha (TNF-α), and interleukin 1β (IL-1β) levels were significantly increased, and the content of glutathione (GSH), activities of superoxide dismutase (SOD), catalase (CAT), alanine aminotransferase (ALT), and aspartate aminotransferase (AST), as well as levels of serotonin and dopamine, were decreased in brain tissues only in AlCl_3_ treated mice. However, treatment of mice with myco-fabrication of ZnO-NPs at doses of 5 or 10 mg/kg improves learning and memory function through ameliorate all the previous parameters in the AD mice group. The low dose of 5 mg/kg is more effective than a high dose of 10 mg/kg. In accordance with these findings, myco-fabricated ZnO-NPs could enhance memory and exhibit a protective influence against memory loss caused by AlCl_3_.

## Introduction

Alzheimer’s disease (AD) is one of the primary health issues whose prevalence has grown recently throughout the world. In 2015, about 44 million people in the world had AD, and by 2050, it is expected that this number will have doubled (Ngolab et al. 2019). The development of AD was connected with a number of variables, involving oxidative stress-induced neuronal injury, loss of acetylcholine in cholinergic neurons, and the formation of β-amyloid (Aβ) plaques in the cells of the brain (Cheignon et al. 2018). A buildup of particular metals, such as aluminum, can start processes that result in the creation of highly reactive radicals. Its simple entry and maintenance in the brain induce oxidative stress that leads to excessive AchE activity that induces a low level of acetylcholine which is linked to the development of β-amyloid plaques and memory loss in AD patients (Liaquat et al. 2019). Malik et al. (2022) reported that AlCl_3_ induced mouse model of AD characterized by memory loss, elevated expression of β-amyloid and increased acetylcholinesterase activity. Many drugs used for the treatment of AD depend on the inhibition of acetylcholinesterase, such as galantamine, rivastigmine, and donepezil. This allows for prolonging the action of the deficient neurotransmitter in the brain, but these drugs have side effects with extended use, e.g., hepatotoxicity (Joe and Ringman 2019). Therefore, searching for treatments with a high potential to reverse neuronal dysfunction and little risk of side effects and expense will be beneficial. Several studies suggested that natural or metal nanoparticle supplements with antioxidant and anti-inflammatory characteristics could be used to regulate oxidative stress and inflammation in order to slow or stop the development of AD (Szczechowiak et al. 2019; Ayaz et al. 2020).

There are numerous uses for metal nanoparticles (NPs) and their oxides in the domains of medicine, agriculture, and industry (El-Sayed et al. 2020a, b, 2023b). The applicability of the NPs has been greatly enhanced by their reduced size, special physicochemical features, and surface changes (Hussein et al. 2022). Among metal oxide nanoparticles, ZnO-NPs are widely employed in biomedical uses such as drug delivery, antibacterial, anticancer, antioxidant, and wound healing (Gomaa et al. 2022). Zinc is a neuromodulator that carries out a variety of physiological actions (Blakemore and Trombley 2017; Hatab et al. 2022), and it is vital for controlling cell proliferation. Additionally, it functions as a molecular signal for transcription factors and immune cells join in the generation of inflammatory cytokines. According to literature, zinc giving diminishes infection incidence and inflammatory cytokine generation. The capacity of zinc to bind metals, along with its role in the catalysis of Cu/Zn superoxide dismutase, preservation of the protein’s –SH group, and upregulation of metallothionein (MT) production, make it a well-known antioxidant (Jarosz et al. 2017).

ZnO-NPs are prepared using different methods, including physical, chemical, and biological ones (Abdelhakim et al. 2020; Mousa et al. 2021). The chemical and physical routes have a number of disadvantages, such as high cost, the need for high-yield equipment, and the formation of unsafe by-products that could be harmful to human health or the environment (Suntako 2015). The green synthesis method eliminates all of these issues by offering safer, extra cost-effectiveness, and less harm to the environment (Singh et al. 2018; Anwar et al. 2022). In the literature, gamma rays can be used as a physical mutagen to improve microbial cultures and develop over-producers of bioactive substances with high economic value (Mousa et al. 2021; El-Sayed 2021; El-Sayed et al. 2022a; b, c). Consistent with Mossa and Shameli (2021), Ag-NPs produced using gamma-irradiated synthesis had a stronger antibacterial impact than those created via chemical synthesis.

In this respect, in our previous study by El-Sayed et al. (2023a), in vitro, we found that the myco-fabricated ZnO-NPs revealed excellent in vitro biological activities, including anti-inflammatory and acetylcholinesterase inhibitory potentials, so we need to apply these results *in vivo.* Thus, the aim of this investigation was to determine whether two distinct doses of ZnO-NPs had any positive effects on biochemical variables related to neurotransmission, oxidative stress, and inflammation in mice that had been given AlCl_3_ to cause AD.

## Materials and methods

### Animals

A total of 60 male albino mice, each weighing 50–60 g and being 9–10 weeks old, were used in the tests. The mice were acclimated before the experiment by spending a week living in our animal building, eating a standard mouse meal, and having unlimited access to water. The mice were separated into six groups equally. The research protocol with serial number 52 A/22 for the purpose of overseeing and monitoring experimental animals was approved by the National Centre for Radiation Research and Technology’s Research Ethics Committee.

### Chemicals

Aluminum Chloride (AlCl_3_, 133.34 M.wt) was bought from Sigma-Aldrich Co., Munich, Germany, and dissolved into distilled water. ZnO-NPs were produced using the gamma-irradiated mutant fungus *Alternaria tenuissima* AUMC10624, according to El-Sayed et al. (2023a). In brief, the was cultured in potato-dextrose broth and the obtained cell-free culture filtrates were mixed with an aqueous solution of the corresponding salt (2 mM zinc sulfate for ZnONPs). Then, the mixture was vigorously stirred for 20 min and kept at room temperature, and the precipitate was separated by ultracentrifugation, washed in deionized water followed by ethanol, and finally dried at 50 °C. Myco-fabricated ZnO-NPs were suspended in deionized water. To prevent particle aggregation, the suspension was well mixed for 1 min before each injection. ZnO nanoparticles mean size of 18.65 nm with a hexagonal crystal structure (El-Sayed et al. 2023a).

### Experimental groups

**Fig. 1:**
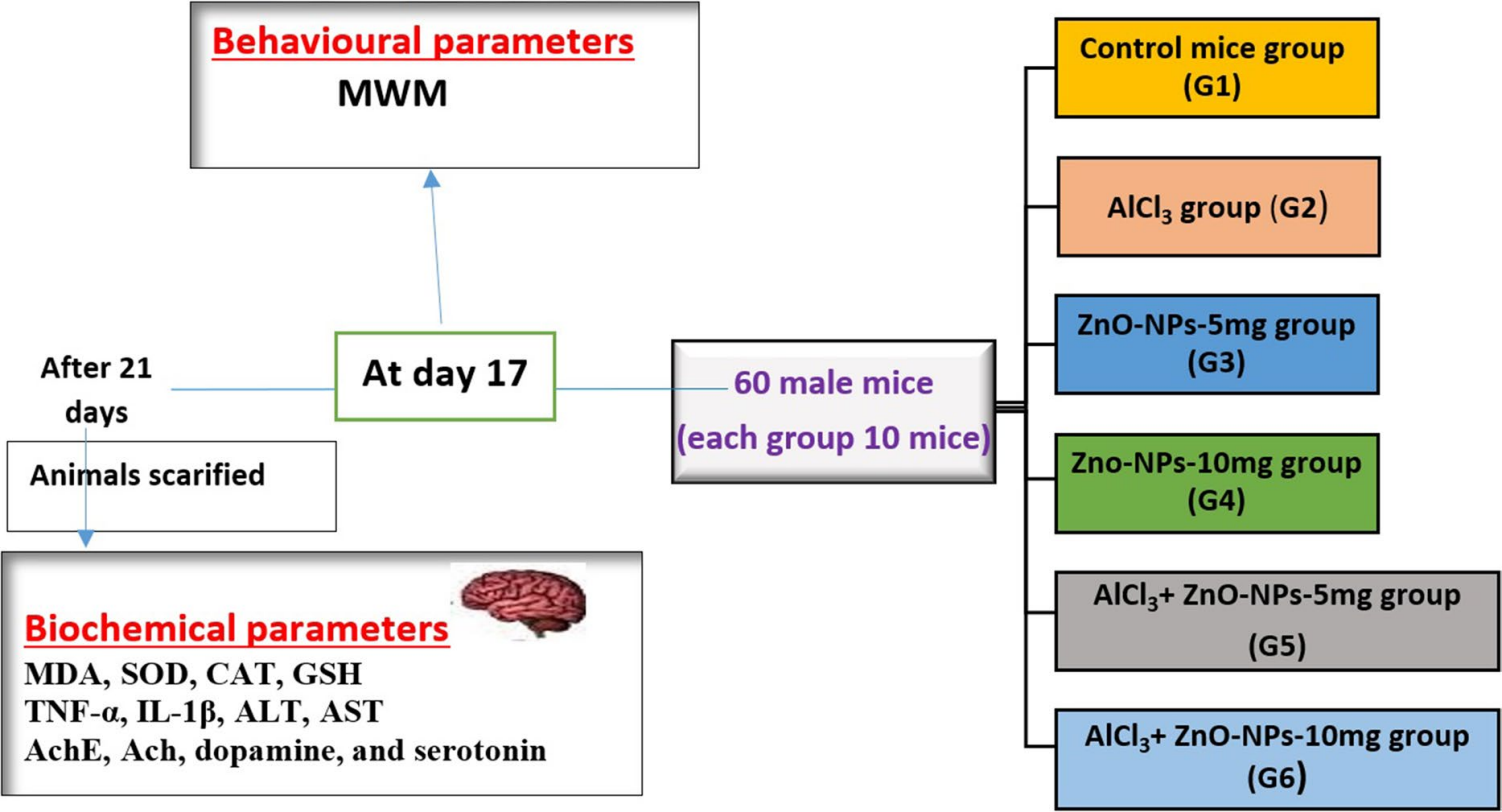
A schematic diagram showing the experimental design

Six groups of mice (Fig. 1) were equally divided as follows:

#### G1:

The control group included mice that were injected i.p. with 0.5 ml of deionized water daily throughout the experiment.

#### G2:

Mice were injected i.p. with AlCl_3_ at a dose of 50 mg/kg to induce AD in mice (Abdelazem 2020) for three weeks.

#### G3 and G4:

Mice were received myco-fabricated ZnO-NPs only (5 and 10 mg/kg /day i.p, respectively) for three weeks daily.

#### G5 and G6 groups:

Included mice were given AlCl_3_ (50 mg/kg i.p.) and afterward treated with myco-fabricated ZnO-NPs (5 mg/kg /day and 10 mg/kg i.p., respectively) 1 h after AlCl_3_ injection for three weeks daily.

### Morris water maze (MWM)

With some modifications to the initial technique, the Morris water maze was utilized in the current investigation to test spatial learning and memory (Vorhees and Williams 2006) in the last week of the experiment. The maze was built as a circular tank with dimensions of 180 cm in diameter and 60 cm in height. It was full of water that was kept at a constant temperature of (27 ± 2 °C) and was made opaque by adding a white nontoxic dye. For the objective of the experiment, four equal quadrants were created in the swimming pool: Northeast, Southeast, Northwest, and Southwest, with one of the diagonal lines serving as the starting point. In the target quadrant, a movable circular platform with a 9 cm diameter was erected on a column and placed in the pool 2 cm below the water’s surface. The first four days of training were spent teaching the animals where the platform was so they were able to attempt to find it. For each mouse to swim in the pool, a cut-off time of 120 s was chosen. Mice were allowed 60 s to locate the platform on the test day (the fifth day) when it was taken away. The total time that the animals consumed in the quadrant of the pool known as the “target” on test day can be used to measure spatial memory (D’Hooge and De Deyn 2001).

### Samples

Once the experimental period has ended, mice were not eating overnight, euthanized with intraperitoneal injections of sodium pentobarbital, and undergoing a complete necropsy. Samples of brain tissue were gathered and then homogenized in 9 volumes of ice–cold 0.05 mM potassium phosphate buffer (pH 7.4) through a glass homogenizer. The supernatant from centrifuging homogenates at 5000 rpm for 15 min at 4 °C was then utilized to measure biochemical parameters.

### Biochemical analysis

#### Brain neurotransmitter biomarkers

Acetylcholine was measured in brain supernatants using a colorimetric choline/acetylcholine assay kit (BioVision Inc., Waltham, MA, USA). The Elisa kits (BioVision) were used to assess the levels of dopamine and serotonin in brain homogenates in accordance with the manufacturer’s instruction.

#### Brain acetylcholinesterase (AchE) activities

The activity of the acetylcholinesterase enzyme was determined using quantification ELISA kits purchased from Cusabio company according to the method of Ellman et al. (1961).

#### Brain lipid peroxidation and antioxidant markers

Using assay kits from Biodiagnostic Co. in Egypt, researchers estimated the levels of glutathione (GSH), malondialdehyde (MDA), catalase (CAT), and superoxide dismutase (SOD) in brain homogenates using the methods of Beutler et al. (1963), Ohkawa et al. (1979), Aebi (1984), and Sun et al. (1988), respectively.

#### Brain inflammation markers

Tumor necrosis factor- alpha (TNF-α) and interleukin 1β (IL-1β) levels in the brain homogenates were quantified using the Ray Bio mouse ELISA technique (Bio-Techne LTD company) in accordance with the manufacturer’s recommendations.

#### Brain enzymes

Diagnostic kits from Biodiagnostic Reagent Kits, Dokki, Giza, Egypt, were used to detect the activity of ALT and AST in the brain supernatant.

### Statistical analysis

The data were displayed as means ± SD. The analysis of difference (ANOVA) in one direction was used. Using the statistical package program COSTAT 3.03,198, Duncan's test was used to compare the groups statistically. At p < 0.05, differences among the groups were measured as significant. The relationship between escape latency and the measured parameters was assessed using Pearson correlation coefficients.

## Results

### Effect of ZnO-NPs on the AlCl^3^ -induced behavioral alterations (memory deficits) in mice by Morris water maze (MWM) test

The MWM test was performed to evaluate the memory and learning ability in mice (for all groups) for 5 days. A two-way analysis of variance was applied to test the significance of the difference between the mean values of escape latency (the actual time it took the mice to reach the platform) of different groups representing the effect of various treatments (A) and time intervals (B). A significant value of A (F = 353.86) and B (F = 529.19) at p < 0.001, were obtained indicating that escape latency differed according to 5 days of training (Table 1). Significant interactions between training days and treatments were also observed (F = 6.57). Duncan^,^s multiple range test revealed AlCl_3_ caused a significant increase in escape latency between time intervals at p < 0.05. while ZnO-NPs alone at two doses showed a significant decrease in the escape latency from day one compared to the control group.

**Table 1:** Effects of ZnO-NPs on escape latency during 5 days intervals in AlCl_3_-stimulated behavioral changes in AD mice

Groups	Escape latency					F and p values		
	Day 1	Day 2	Day 3	Day 4	Day 5	A	B	A and B
G1	54.8 ± 2.3	44 ± 2.28	33.24 ± 1.18	28.4 ± 1.02	24.4 ± 1.01	353.86	529.19	6.57
G2	63.4 ± 2.6	58.6 ± 0.97	49.2 ± 3.05	45.6 ± 3.14	43.6 ± 1.85			
G3	34.4 ± 3.93	31.6 ± 1.88	29.6 ± 1.95	23.8 ± 1.47	18.6 ± 1.85	p ≤ 0.001	p ≤ 0.001	p ≤ 0.001
G4	49.2 ± 1.72	41.4 ± 3.07	32.0 ± 1.38	28.8 ± 1.16	23.6 ± 2.94			
G5	58.0 ± 2.09	50.4 ± 1.02	39.4 ± 1.01	33.6 ± 1.62	31.4 ± 1.49			
G6	61.0 ± 1.22	54.8 ± 3.2	47.4 ± 1.85	39.2 ± 2.04	33.8 ± 1.93			

*F* to-way analysis of variance,* A* comparison among the treatment,* B* comparisons among the time intervals,* A and B* the interaction between treatment and times

**Fig. 2:**
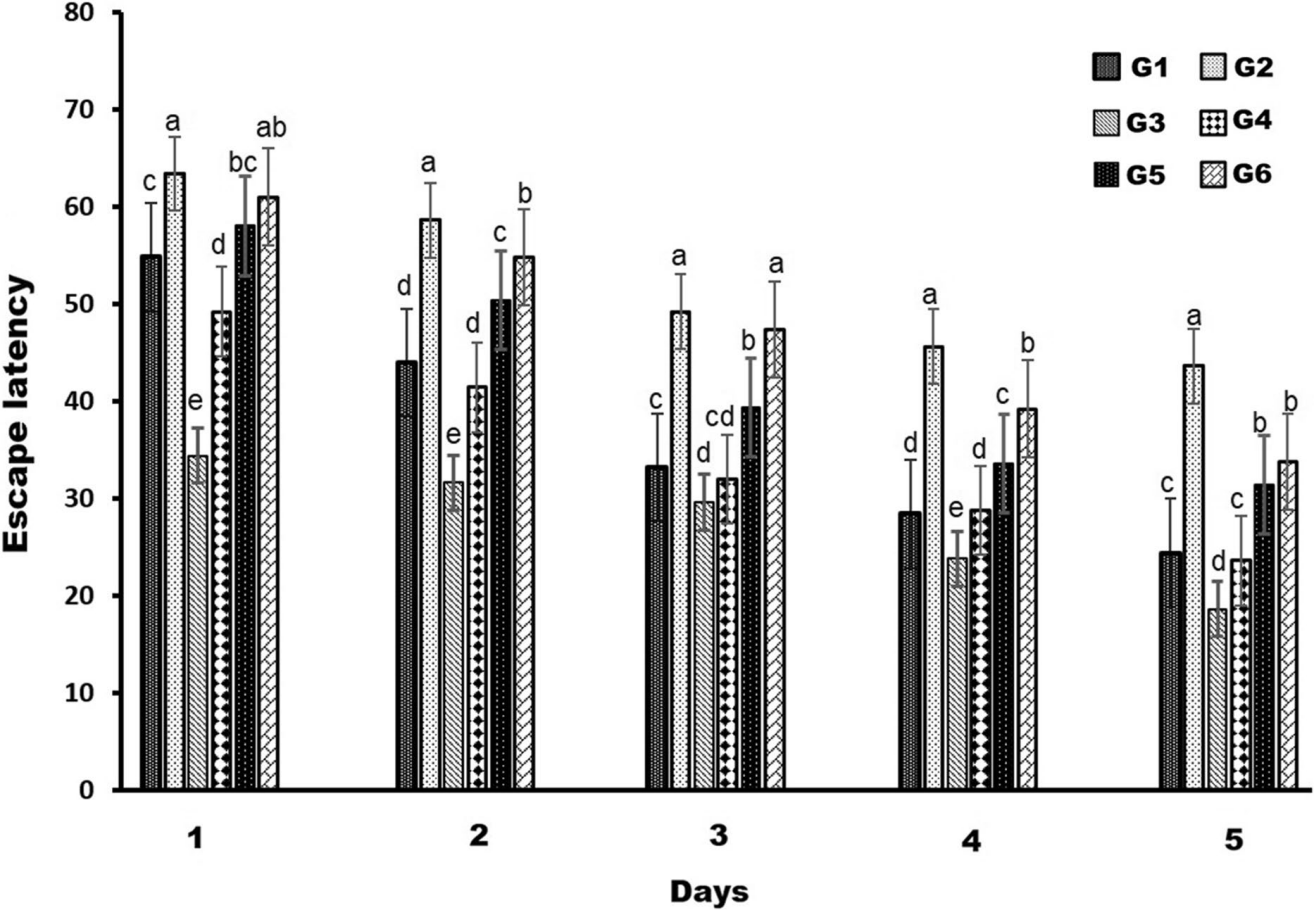
The MWM test was used to determine the effects of ZnO-NPs on escape latency in AlCl_3_-stimulated behavioral changes in AD mice. The results were provided as mean ± SD (one-way ANOVA followed by Duncan's test). A distinct superscript is used to denote a significant difference (p ≤ 0.05) in comparison to the control group. a–e means with different superscripts for each data series are considered significantly different

**Fig. 3:**
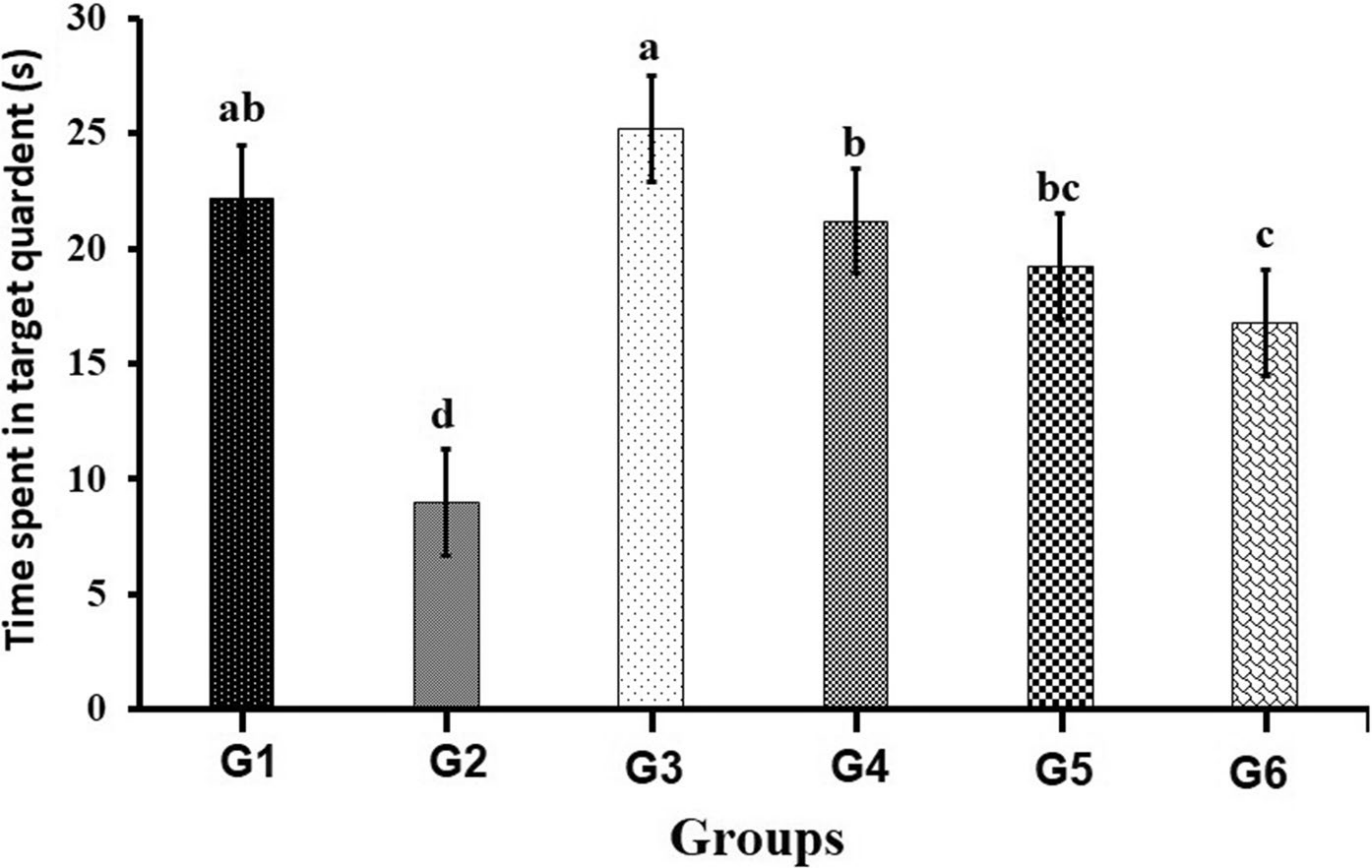
Results of the MWM test measuring the effects of ZnO-NPs on the total time spent in the target quadrant in AlCl_3_-stimulated behavioral changes in AD mice were shown as mean ± SD one-way ANOVA followed by Duncan's test). A distinct superscript is used to denote a significant difference (p ≤ 0.05) in comparison to the control group. a–d means with different superscripts for each data series are considered significantly different

Additional analysis with one–way ANOVA showed that mice given AlCl_3_ demonstrated a substantial (p < 0.05) increase in escape latency and a decrease in time spent in the target quadrant compared to the control group. In comparison to the AlCl_3_ group, the mice who were co-treated by ZnO-NPs (5 and 10 mg/kg) had significantly shorter travel times to the platform and spent extra time in the desired quadrant (Figs. 2 and 3). These findings demonstrate that mice given AlCl_3_ may recover spatial memory by giving ZnO-NPs. Furthermore, in comparison to the control group, mice that were given ZnO-NPs alone showed statistically significant elevations in time spent in the desired quadrant and reductions in escape latency, 5 mg/kg ZnO-NPs being more efficient than 10 mg/kg ZnO-NPs.

### Effect of myco-fabricated ZnO-NPs on variations in lipid peroxidation and antioxidant indicators in the brain of mice

There were significant reductions (p < 0.05) in the levels of GSH (97.2 ± 4.09) in addition to activities of CAT (132.0 ± 1.58) and SOD (143.2 ± 8.53) in the brain tissues of mice treated by AlCl_3_ compared to control (127.2 ± 4.4, 288.0 ± 13.04 and 216.6 ± 3.85, respectively) (Table 2). Moreover, there was a significant (p ≤ 0.05) elevation in brain levels of MDA (10.04 ± 0.35) in AlCl_3_-intoxicated mice compared to control values (6.46 ± 0.31). In contrast, all treated animals, notably those given 5 mg/kg of myco-fabricated ZnO-NP alone showed a statistically significant reduction in brain MDA levels (4.19 ± 0.34) and increases in GSH content (176.4 ± 5.03), as well as CAT (489.1 ± 5.83) and SOD (285.0 ± 6.21) activity compared to the control group. These findings imply that ZnO-NPs at a concentration of 5 mg/kg are more effective for reducing oxidative stress in the mouse brain.

**Table 2:** Effect of myco-fabricated ZnO-NPs on lipid peroxidation and antioxidant indicators in the brain of mice administrated with AlCl_3_.

Parameters	Groups						F value	p value
	G1	G2	G3	G4	G5	G6		
MDA (nmol/mg)	6.46 ± 0.31^b^	10.04 ± 0.35^a^	4.19 ± 0.34^d^	5.08 ± 0.24^c^	5.14 ± 0.44^c^	6.38 ± 0.39^b^	177.80	≤ 0.001
% of changes		55.41	−35.14	−21.36	−20.43	−1.23		
SOD (U/mg)	216.6 ± 3.85^c^	143.2 ± 8.53^f^	285.0 ± 6.21^a^	236.1 ± 3.94^b^	173.2 ± 3.63^d^	166.4 ± 4.39^e^	523.5	≤ 0.001
% of changes		−33.89	31.57	9.0	−20.04	−23.18		
CAT (ng/mg)	288.0 ± 13.04^c^	132.0 ± 1.58^f^	489.1 ± 5.83^a^	352.2 ± 6.91^b^	158.4 ± 6.11^d^	143.2 ± 2.59^e^	920.8	≤ 0.001
% of changes		−54.17	69.83	22.29	−45	−50.28		
GSH (ng/mg)	127.2 ± 4.4^c^	97.2 ± 4.09^e^	176.4 ± 5.03^a^	156.6 ± 3.98^b^	122.8 ± 2.59^c^	102.4 ± 3.05^d^	307.13	≤ 0.001
% of changes		−23.58	38.68	23.11	−3.46	−19.49		

The mean and standard deviation of ten mice from each group are used to represent the values. Means with different superscripts within the same row differ significantly at (p  ≤ 0.05)

### Effect of ZnO-NPs on alteration of pro-inflammatory cytokines and transaminases enzymes in the brain of mice

Mice that were given AlCl_3_ resulted in a statically significant rise (p ≤ 0.05) of the pro-inflammatory cytokines TNF- α (287.0 ± 4.53) and IL-1 β (418.0 ± 23.87) as well as a significant reduction in the activities of AST (11.4 ± 0.14) and ALT (3.6 ± 0.32) in brain cells in comparison to mean values of control mice (74.4 ± 2.51, 282.0 ± 14.83, 34.0 ± 0.36 and 11.86.0 ± 0.85, respectively). In AlCl_3_-intoxicated mice, a combined treatment with myco-fabricated ZnO-NPs at two doses (5 and 10 mg/kg) resulted in a significant decrease (p ≤ 0.05) in TNF- α (108.0 ± 7.97, 124.8 ± 3.89) and IL-1 β levels (336.0 ± 23.02, 384.0 ± 15.16) and an increase in AST (27.2 ± 0.16, 16.4 ± 0.29) and ALT (8.86 ± 0.98, 5.28 ± 0.77) activities in comparison to animals treated with AlCl_3_ alone, but these values were still significantly different (p ≤ 0.05) from the control values. The mice treated with 5 mg/kg myco-fabricated ZnO-NPs rather than 10 mg/kg ZnO-NPs showed the best outcomes. Additionally, in myco-fabricated ZnO-NPs alone at the two doses in comparison to the control group, IL-1 β was considerably reduced (p ≤ 0.05). Comparing mice treated with ZnO-NPs alone to control mice, the levels of TNF-α and the activities of ALT and AST did not alter significantly (Table 3).

**Table 3:** Effect of myco-fabricated ZnO-NPs on changes in pro-inflammatory cytokines and transaminase enzymes in the brain of mice induced by AlCl_3_

Parameters	Groups						F value	p value
	G1	G2	G3	G4	G5	G6		
TNF- α (pg/ml)	74.4 ± 2.51^d^	287.0 ± 4.53^a^	75.6 ± 4.83^d^	76.4 ± 8.21^d^	108.0 ± 7.97^c^	124.8 ± 3.89^b^	1037.02	≤ 0.001
% of changes		285.7	1.61	2.69	45.16	67.74		
IL1B (pg/ml)	282.0 ± 14.83^d^	418.0 ± 23.87^a^	225.0 ± 11.18^e^	236.8 ± 17.06^e^	336.0 ± 23.02^c^	384.0 ± 15.16^b^	94.92	≤ 0.001
% of changes		48.23	−20.21	−16.03	19.15	36.17		
AST(U/L)	34.0 ± 0.36^a^	11.4 ± 0.14^d^	33.0 ± 0.21^a^	34.4 ± 0.32^a^	27.2 ± 0.16^b^	16.4 ± 0.29^c^	74.29	≤ 0.001
% of changes		−66.47	−2.94	1.18	−20	−51.76		
ALT (U/L)	11.86.0 ± 0.85^a^	3.6 ± 0.32^d^	12.20 ± 1.68^a^	10.98 ± 0.58^a^	8.86 ± 0.98^b^	5.28 ± 0.77^c^	70.41	≤ 0.001
% of changes		−69.65	2.86	−7.41	−25.29	−55.48		

The mean and standard deviation of ten mice from each group are used to represent the values. Means with different superscripts within the same row differ significantly at (p ≤ 0.05)

### Effect of myco-fabricated ZnO-NPs on changes in acetylcholinesterase, acetylcholine, dopamine, and serotonin in the brain of mice test

Table 4 shows how ZnO-NPs affect acetylcholinesterase, acetylcholine, dopamine, and serotonin. A statistically significant (p < 0.05) decline in the levels of Ach (0.91 ± 0.14), dopamine (12.4 ± 0.52), and serotonin (49.02 ± 2.35) was found in the AlCl_3_ group when compared to the normal group (6.0 ± 0.36, 33.46 ± 2.72 and 116.76 ± 4.64, respectively). Additionally, compared to control mice (0.36 ± 0.04), a substantial (p < 0.05) rise in acetylcholinesterase activity was found in mice treated with AlCl_3_ (1.91 ± 0.14). The values of all the earlier parameters almost reverted to the control value when mice were treated with ZnO-NPs at two doses (5, 10 mg/kg). The results from the lower dose of ZnO-NPs (5 mg/kg) are better to those from the higher dose (10 mg/kg).

**Table 4:** Effect of myco-fabricated ZnO-NPs on changes in acetylcholinesterase, acetylcholine, dopamine, and serotonin in the brain of mice induced by AlCl_3_

Parameters	Groups						F value	p value
	G1	G2	G3	G4	G5	G6		
AchE (pg/mg)	0.36 ± 0.04^d^	1.91 ± 0.14^a^	0.39 ± 0.02^d^	0.38 ± 0.03^d^	0.53 ± 0.02^c^	0.62 ± 0.04^b^	436.08	≤ 0.001
% of changes		430.56	8.33	5.56	47.22	72.22		
Ach (pg/mg)	6.0 ± 0.36^a^	0.91 ± 0.14^d^	6.34 ± 0.21^a^	5.76 ± 0.32^a^	5.0 ± 0.16^b^	4.46 ± 0.29^c^	288.9	≤ 0.001
% of changes		−84.83	5.67	−4	−16.67	−25.67		
Dopamine (ng/mg)	33.46 ± 2.72^a^	12.4 ± 0.52^c^	35.0 ± 1.19^a^	33.6 ± 0.37^a^	34.12 ± 3.69^a^	21.76 ± 0.97^b^	107.94	≤ 0.001
% of changes		−62.94	4.60	0.42	1.97	−34.96		
Serotonin (ng/mg)	116.76 ± 4.64^a^	49.02 ± 2.35^c^	105.7 ± 7.11^a^	96.0 ± 3.54^ab^	93.0 ± 5.09^ab^	69.66 ± 5.38^bc^	121.06	≤ 0.001
% of changes		−58.02	−9.47	−17.78	−20.35	−40.34		

The mean and standard deviation of ten mice from each group are used to represent the values. Means with different superscripts within the same row differ significantly at (p  ≤ 0.05)

### The correlation between escape latency and some brain biochemical parameters

Table 5 shows correlation coefficient (r) values between escape latency and some brain biochemical parameters. There was a significant positive correlation between escape latency and MDA (r = 0.7346, p < 0.01), AchE (r = 0.8552, p < 0.01), TNF-α (r = 0.8837, p < 0.01), IL 1ß (r = 0.9171, p < 0.01). Moreover, there was a negative correlation between escape latency and GSH (r = − 0.7018, p < 0.01), CAT (r = − 0.7041, p < 0.01), SOD (r = − 0.800, p < 0.01), ST (r = − 0.7112, p < 0.01), DA (r = − 0.9056, p < 0.01), Ach (r = − 0.9075, p < 0.01), ALT (r = − 0.9023, p < 0.01) and AST (r = − 0.8905, p < 0.01).

**Table 5:** Correlation coefficient between escape latency and biochemical parameters in the brain of mice

Brain parameters	Escape latency
GSH	− 0.7018
CAT	− 0.7041
SOD	− 0.80
MDA	0.73463
ST	− 0.7112
DA	− 0.9056
AchE	0.8552
Ach	− 0.9075
ALT	− 0.9023
AST	− 0.8905
TNF-α	0.88375
IL-1β	0.91712

## Discussion

The present study proved the ameliorative action of myco-fabricated ZnO-NPs against AlCl_3_-induced AD in mice. Aluminum cross into the brain through the specific great affinity receptors for transferrin expressed in the blood–brain barrier (Roskams and Connor 1990). The hippocampus and cortex are crucial for cognitive functions including learning and memory, and these areas are the most susceptible to AD and Al poisoning (Malik et al. 2022). In the current research, supplementation of Myco-fabricated ZnO-NPs significantly ameliorated the neural, behavioral, and biochemical abnormality in AlCl_3_-induced AD in mice, which means the beneficial and neuroprotective action of Myco-fabricated ZnO-NPs against the AD. Baydar et al. (2003) stated that the measurements of behavioral alterations are more sensitive than neurochemical variations as signs of neurotoxicity through AlCl_3_ exposure. According to our current findings, mice treated with AlCl_3_ had poorer spatial memory and accuracy, as shown by higher escape latencies to the platform and less time spent on the target (platform) quadrant during the MWM test. This may be due to the accumulation of aluminum in the brain, which induces increasing AchE activity, inflammation, and the accumulation of beta-amyloid, as well as reducing the antioxidant activity that affects learning and memory (Thenmozhi et al. 2015). This behavioral alteration is confirmed by the biochemical changes in the AlCl_3_ groups compared with the normal group. Our study findings were in agreement with the previous article stated by Ekundayo et al. (2022). However, myco-fabricated ZnO-NPs treatment at two different doses significantly enhanced this diminished spatial learning and memory adjacent to the control group, and the dose of 5 mg/kg/bw was more powerful than 10 mg/kg/bw. This may be related to zinc having antioxidant, and anti-inflammatory action (Jarosz et al. 2017). These findings suggest that myco-fabricated ZnO-NPs has a memory improving function and a protective effect against AlCl_3_-induced memory loss through antioxidant, anti-inflammatory, and inhibitory actions for AchE.

In the current investigation, mice treated with AlCl_3_ displayed substantial changes in brain MDA concentration, GSH content, SOD, and CAT activity, all of which are markers of enhanced oxidative damage and lipid peroxidation caused by aluminum accumulation in brain tissues. Due to its high oxygen consumption and insufficient antioxidant system, the brain is particularly vulnerable to oxidative stress (Parashar and Udayabanu 2017). Thus, neurotoxicity caused by AlCl_3_ might be due to the overproduction of ROS resulting in considerable neuronal injury arising from disorders in the antioxidant defense system. It is widely known that aluminum can enter the blood-brain barrier and build up in many brain tissues, promoting the production of free radicals, which in turn raises protein and DNA oxidation and lipid peroxidation. Aluminum has also been demonstrated to interfere with iron homeostasis by displacing iron from the iron transport protein transferrin, increasing the amount of redox-active iron in brain tissues (Vieelien et al., 2022). This causes significant oxidative damage and may result in brain injury, particularly in regions of the brain associated with memory and learning (Saba et al. 2017). Khan et al. (2011) found that the significant decrease in GSH in the AlCl_3_-treated group may have resulted from aluminum attaching to the SH group of GSH, which can be excreted, reducing GSH’s ability to act as a neutrophilic scavenger. Nehru and Anand (2005) observed that decreased activities of SOD and CAT in rat’s brains exposed to AlCl_3_ may be attributed to a decrease in the synthesis of enzyme proteins. Similarly, numerous studies stated that declines in activities of SOD and CAT are connected with AD (Jadhav and Kulkarni 2022; Ekundayo et al. 2022; Ojha 2023 ; Chen et al. 2021).

One of the potential components that can stop AD from starting and progressing is antioxidants. The mice treated with myco-fabricated ZnO-NPs alone had higher levels of GSH as well as activities of SOD and CAT than the other groups in the current study. This may be attributable to an increase in Zn concentration in the brain tissue as a result of Zno nanoparticle dissociation. According to Sidhu and Garg (2005), zinc reduces the action of pro-oxidant enzymes, inhibits lipid peroxidation, and promotes the production of proteins and enzymes such as antioxidant proteins, GSH, CAT, and SOD. According to Abd Elmonem et al. (2021) ZnO-NPs can decrease MDA levels, improve antioxidant enzyme activities, and protect cell membrane integrity from oxidative stress damage. Furthermore, Zhao et al. (2014) verified that Cu-Zn-SOD activity is stimulated by a suitable concentration of ZnO-NPs, and this improvement will reduce ROS production. Hence, myco-fabricated ZnO-NPs (5 or 10 mg/kg) given to the AlCl_3_ group revealed a significant reduction in the brain MDA and significantly improved SOD and CAT activities as well as GSH levels compared with the AlCl_3_ treated group. Additionally, a dose of 5 mg/kg of myco-fabricated ZnO-NPs was more efficient than a dose of 10 mg/kg, meaning that a low dose of these particles had powerful antioxidant effects by increasing antioxidant activity and lowering free radical levels.

Numerous authors have studied the connection between oxidative stress and inflammation, and they discovered that high levels of pro-inflammatory cytokines are associated with low antioxidant levels and insufficient antioxidant enzyme activity (Salim et al. 2012). Our findings indicated an increase in brain TNF-α and IL-1β, which may be related to an increase in oxidative stress induced by aluminum in the AlCl_3_ group. According to Popa-Wagner et al. (2013), ROS produced in brain cells can alter synaptic and non-synaptic transmission among neurons, leading to neuro-inflammation, cell death, neuro-degeneration, and memory loss. Previous studies have shown that neuro-inflammatory cytokines reduce the efflux transfer of amyloid (A*β)*, which results in increased A*β* concentrations in the brain (Blasko et al. 1999). Amyloid β plaque formation in the brain is one of the primary causes of AD (Murphy and LeVine 2010). Additionally, it is shown that TNF-α plays an essential role in A*β* made destruction of LTP, a kind of synaptic plasticity directly related to memory and learning (Wang et al. 2005).

In the current investigation, it was found that myco-fabricated ZnO-NPs co-treatment with AlCl_3_ decreased the rise levels of TNF-α and IL-1β in mice^’^s brains in comparison with AlCl_3_ alone, which significantly raised these cytokines. This may be attributed to the elevated Zn content in the brain tissue. Zinc improves the up-regulation of A20 protein (TNF-α-induced protein 3). It is a highly conserved protein that has seven zinc finger (ZnF) domains in its C-terminus, which decline NF-kappaB activation, causing reduced gene expression and the generation of TNF-α, IL-1 ß, and IL-8 (Dardenne 2002; Prasad 2008). This result confirms our previous study in vitro, which showed that myco-fabricated ZnO-NPs have wound healing, anti-inflammatory action (El-Sayed et al. 2023a, b). Our findings concur with earlier research that showed the ability of ZnO-NPs to reduce inflammation (Ekundayo et al. 2022; Chen et al. 2021; Abdulmalek et al. 2021).

Aspartate aminotransferase and alanine aminotransferase are active brain enzymes that are found in cytosolic and mitochondria and they have a role in glutamate metabolism (Palailogos et al. 1989). The significant decline in brain AST and ALT activities in the AlCl_3_ group could be related to oxidative stress formed from the buildup of aluminum in brain tissues, which disturbs protein synthesis and causes a decrease in ALT and AST activities (Netopilová et al. 2001). The decline in transaminase enzymes indicates a decrease in glutamate metabolism, causing neurological dysfunction. Glutamate plays a part in synaptic plasticity, one of the key neurochemical bases of memory and learning, which is important for cognitive processes like memory and learning in the brain (Meldrum 1994). Bartos et al. (2019) reported that oxidative stress induced by exposure to fluoride caused a decline in ALT and AST enzymes in brain offspring rats, which led to a decrease in glutamate, a possible mechanism of neurotoxicity and memory impairment. Moreover, Amel et al. (2016) found that giving rats 1000 ppm lead acetate in drinking water decreased brain ALT and AST. Moreover, myco-fabricated ZnO-NPs ameliorated these enzymes, which may be related to the antioxidant effects of myco-fabricated ZnO-NPs.

The current investigation found that AlCl_3_ significantly increased AchE activity and decreased Ach levels. This might be related to aluminum’s allosteric interaction with the peripheral anionic site of the enzyme molecule (Pohanka 2011), producing variation of its secondary structure and so increasing its activity **(**Zatta et al. 1994). An additional explanation for the elevated AchE could be related to IL-1ß overproduction, which stimulates the activity and expression of AchE through the interaction of IL-1β by muscarinic acetylcholine receptors **(**Schliebs et al. 2006). Also, an increase in AchE may be due to increased oxidative stress and lipid peroxidation and a decrease in antioxidant capacity induced by aluminum in brain tissues (Kumar and Gill 2014). Kaizer et al. (2005) suggested that changes in the lipid membrane might be responsible for an alteration in the structural form of the AchE molecule, that make induction of AchE activity after prolonged exposure to aluminum. Additionally, the levels of the neurotransmitter dopamine and serotonin (5-hydroxytryptamine, 5-HT) in the mice’s brain tissue significantly decreased in the AlCl_3_-exposed animals. This could be linked to the oxidative stress caused by AlCl_3_, which makes the oxidation of tryptophan a precursor to 5-HT. It is true that both ROS and pro-inflammatory cytokines can convert tryptophan to kynurenine. According to Bakunina et al. (2015), this molecule might be further metabolized to the pro-oxidant substances 3-hydroxykynurenine and quinolinic acid, which are linked to the causing of depression. Also, Cunnington and Channon (2010) illustrated that an increase in ROS can lead to reduced availability of tetrahydrobiopterin (BH4). BH4 is a cofactor that uses the three aromatic amino acid hydroxylase enzymes to produce the precursors of the major monoamine neurotransmitters dopamine and serotonin from aromatic amino acids like phenylalanine, tyrosine, and tryptophan. This reduced synthesis of dopamine and serotonin results from the availability of these precursors (Kappock et al.  1996). The results of our investigation concurred with those of the earlier study mentioned by Ekundayo et al. (2022).

We found that the AchE activity in brain tissues was significantly suppressed in both doses of myco-fabricated ZnO-NP (5 and 10 mg/kg)-treated mice, which confirms our previous study in vitro by El-Sayed et al. (2023a,) we found that myco-fabricated Zno-Ps have a stronger inhibitory influence on AchE through direct interaction with AchE by molecular docking. Inhibited AchE activity can prevent Ach from being degraded in the synaptic cleft, resulting in a buildup of Ach, that augments cholinergic neurotransmission and improves memory and cognition in animals. This result suggested that myco-fabricated ZnO-NPs has an ameliorating effect on neurodegenerative symptoms in AD through its antioxidant, anti-inflammatory, and inhibiting AchE. Thus, ZnO-NPs can be enhancing cognition in experimental animals by elevating acetylcholine at synapses. The anti-cholinesterase activities ZnO-NPs detected in our research are in alignment with previous studies (Guo et al. 2020; Hamza et al. 2019). According to Lu et al. (2013), zinc was found to mitigate the negative effects of aluminum exposure on AchE activity, dopamine and serotonin levels, and brain redox status.

Longer escape latencies to arrive at the platform and less time spent in the target quadrant during the MWM test in AlCl_3_-treated mice indicate deteriorating spatial memory, which is indicative of poor learning and memory. This may be due to the accumulation of aluminum in the brain, which induces increasing AchE activity, inflammation, and the accumulation of beta-amyloid, as well as reducing the antioxidant activity that affects learning and memory (Thenmozhi et al. 2015). These behavioral results supported the previous biochemical finding. Myco-fabricated ZnO-NP treatment at two different doses significantly enhanced this diminished spatial learning and memory adjacent to the control group, and the dose of 5 mg/kg/bw was more powerful than 10 mg/kg/bw. These findings suggest that myco-fabricated ZnO-NPs have a memory-improving function and a protective effect against AlCl_3_-induced memory loss through antioxidant, anti-inflammatory, and inhibitory actions for AchE.

## Conclusion

The findings of this work demonstrated that myco-fabricated ZnO-NPs provide neuro-amelioration against an experimental AD model caused by AlCl_3_ through reducing IL1 β, TNF-ἀ, MDA, and activity of AChE and increasing production of GSH, SOD, and CAT. Also, myco-fabricated ZnO-NPs can enhance behavioral alterations by reducing escape latency and increasing time spent in the target quadrant. This indicates that the myco-fabricated ZnO-NPs (especially 5 mg/kg b.w) have antioxidant, anti-inflammatory, and inhibitory action for AChE, and these in vivo results confirm our previous study in vitro.

## Abbreviations

Ach: Acetylcholine
AchE: Acetylcholinesterase
ALT: Alanine aminotransferase
AST: Aspartate aminotransferase
CAT: Catalase
DA: Dopamine
GSH: Glutathione
IL-1β: Interleukin-β
MDA: Malondialdehyde
SOD: Superoxide dismutase activity
ST: Serotonin
TNF-α: Tumor necrosis factor-alpha
